# Adaptive dynamic prediction model of mining subsidence aided by measured data

**DOI:** 10.1038/s41598-025-99869-x

**Published:** 2025-04-28

**Authors:** Yuanfei Chen, Jianfeng Zha, Lei Wang

**Affiliations:** 1 School of Geography and Planning, Chizhou University, Chizhou, 247000 Anhui China; 2 School of Environment and Spatial Informatics, China University of Mining and Technology, Xuzhou, 221116 Jiangsu China; 3 School of Earth and Environment, Anhui University of Science and Technology, Huainan, 232001 Anhui China; 4 School of Spatial Information and Geomatics Engineering, Anhui University of Science and Technology, Huainan, 232001 Anhui China

**Keywords:** Environmental sciences, Natural hazards, Energy science and technology

## Abstract

Underground mining-induced surface subsidence adversely affects both the surface environment and the structures located above it. Accurately predicting the dynamic subsidence and deformation caused by underground mining is crucial when employing maintenance and remediation methods to mitigate these adverse effects, as it directly impacts the selection of maintenance strategies, timing, and volume assessments. In response to the limitations of traditional time function and parameter models in adapting to the dynamic changes of actual underground mining activities—resulting in low subsidence prediction accuracy—this paper presents an adaptive prediction model for dynamic subsidence supported by measured data and developed through programming. This model utilizes historically measured data on surface subsidence to derive optimal parameters for each historical period. By analyzing the trends in these parameters, it dynamically adjusts the parameter value for subsequent predictions, achieving high-precision prediction of the surface dynamic subsidence. Engineering case study results indicate significant variations in the optimal time function parameter values throughout the mining process. The estimated parameter values obtained through the extrapolative prediction method, supported by measured data, align closely with the optimal values. The average relative RMSE of predicted dynamic subsidence for each period is 4.3%, markedly lower than the 9.1% achieved by traditional prediction models. This enhancement significantly improves the accuracy of dynamic subsidence predictions due to underground mining and provides robust technical support for the maintenance and remediation of structures.

## Introduction

Coal is one of the most basic energy sources worldwide. In 2023, coal energy consumption accounted for 26% of the global energy structure, with China’s coal consumption representing 56% of the world’s total^1^. To meet the demand for coal resources in China’s modernization process, more and more coal mines have conducted coal mining beneath villages, buildings, roads, and water bodies. The overburden and surface subsidence caused by underground mining have adverse effects on the ground and various structures above it, such as buildings, railways, highways, and high-voltage transmission lines. In severe cases, this can lead to a range of different disasters^2–4^. Generally, methods such as relocation, reinforcement, or maintenance are employed to mitigate the impact of these adverse disasters on surface structures^5–7^. When adopting reinforcement or maintenance measures to address the adverse effects of subsidence, selecting appropriate maintenance methods, determining the right timing for repairs, and accurately estimating the volume of maintenance work all rely on high-precision dynamic predictions of surface subsidence to provide the necessary data support.

The current methods for dynamic predictions of surface subsidence mainly include time series methods, numerical simulation methods, and influence function methods. The time series method posits that surface dynamic subsidence follows a specific time series. This time series is theoretically modeled, allowing for dynamic predictions of surface subsidence using the established mathematical model. This method finds it challenging to account for the impact of the underground mining process, and when factors such as advancing speed, coal seam thickness, and coal seam depth change, the predicted results often exhibit significant deviations. The numerical simulation method has the advantages of repeatability and low cost^8^. Although some literature^9–12^ has explored rock mechanical parameters and their failure characteristics, the challenge of accurately determining the mechanical parameters and constitutive models of rock layers in numerical simulations still limits the application of this method in quantitative calculations. The influence function method essentially determines the impact of small unit mining on overburden or the surface based on theoretical research or other methods. It considers the overall impact of an entire working face on the surface as the sum of the impact caused by multiple small unit mines, and computes the surface movement and deformation caused by the mining of the entire working face accordingly^13^. The probability integral model(PIM) is an important model within the influence function method. This approach has been incorporated into industry regulations and has seen widespread application in China^14^. By combining it with time function models, such as the Knothe model^15^, Logistic curve model^16^, Weibull curve model^17^, and so on^18–21^, the dynamic subsidence value of the surface can be calculated at any time. The Knothe time function is widely used due to its solid mathematical foundation and physical significance. However, this model cannot accurately reflect the movement process of surface points affected by underground mining activities^22^, making it difficult to determine the exact value of the function parameter and, consequently, affecting the accuracy of dynamic predictions.

This paper addresses the issue of inaccurate parameter in the Knothe time function, which results in low accuracy in predicting dynamic subsidence on the surface. Considering the engineering context, where significant surface structures (such as ultra-high voltage transmission towers, highways, and high-speed railways) require frequent monitoring, prediction, and maintenance during the mining period to ensure safety, we propose an adaptive dynamic subsidence prediction model aided by measured data and implement it through programming. The model utilizes historical monitoring data from previous subsidence to automatically adjust the parameter in the Knothe time function, thereby improving the adaptability of the traditional model and the accuracy of the predicted subsidence. Based on the practical application of the coal mining project under the high-grade highway at Nantun Coal Mine, the accuracy and reliability of the method were validated.

## Dynamic prediction model of surface subsidence

### Surface subsidence prediction principles by PIM method

**Fig. 1:**
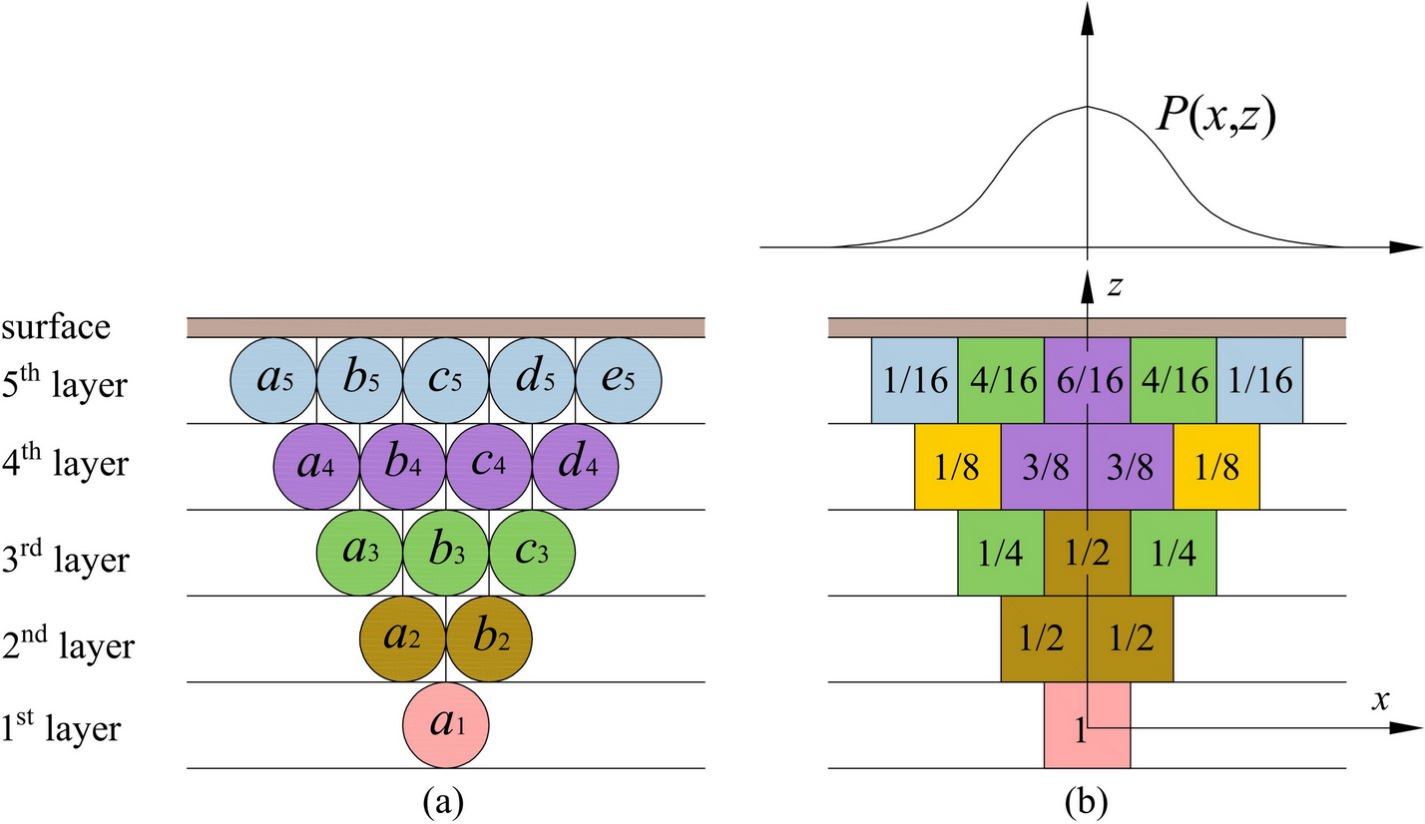
Theoretical model of PIM. (**a–e**) Represent different rock particle units.

The probability integral model (PIM) is one of the most widely used methods for predicting subsidence caused by mining. It is based on the random medium theory^23^, and considers that the mining-induced overburden and surface movement laws are similar to the laws described by the granular medium model on the macro level. The mining area is divided into small mining units, and the mining-induced surface movement and deformation could be obtained by the accumulating of surface movement and deformation caused by the mining of these micro units^13^. The basic principles of the PIM could be described in Fig. 1.

In this model, after the excavation of the first layer unit $a_{1}$, the second layer units $a_{2}$ and $b_{2}$ each have a probability of 1/2 of moving downward. The third layer units $a_{3}$, $b_{3}$, and $c_{3}$ have probabilities of 1/4, 1/2, and 1/4, respectively, of moving downward. Following this pattern of transmission, the subsidence probabilities for each surface unit can be obtained:

1 $$\begin{aligned} w_{e}(x)=\frac{1}{r} e^{-\frac{\pi x^{2}}{r^{2}}}, \end{aligned}$$

where, $w_{e}(x)$ represents the subsidence value at the point with surface coordinates *x* after the extraction of unit $a_{1}$. *r* represents the main influence radius, its formula is $r=H/\tan \beta$ . *H* is the mining depth of the working face. And the $\tan \beta$ is the tangent of the main influence angle, it’s an important parameter in PIM.

**Fig. 2:**
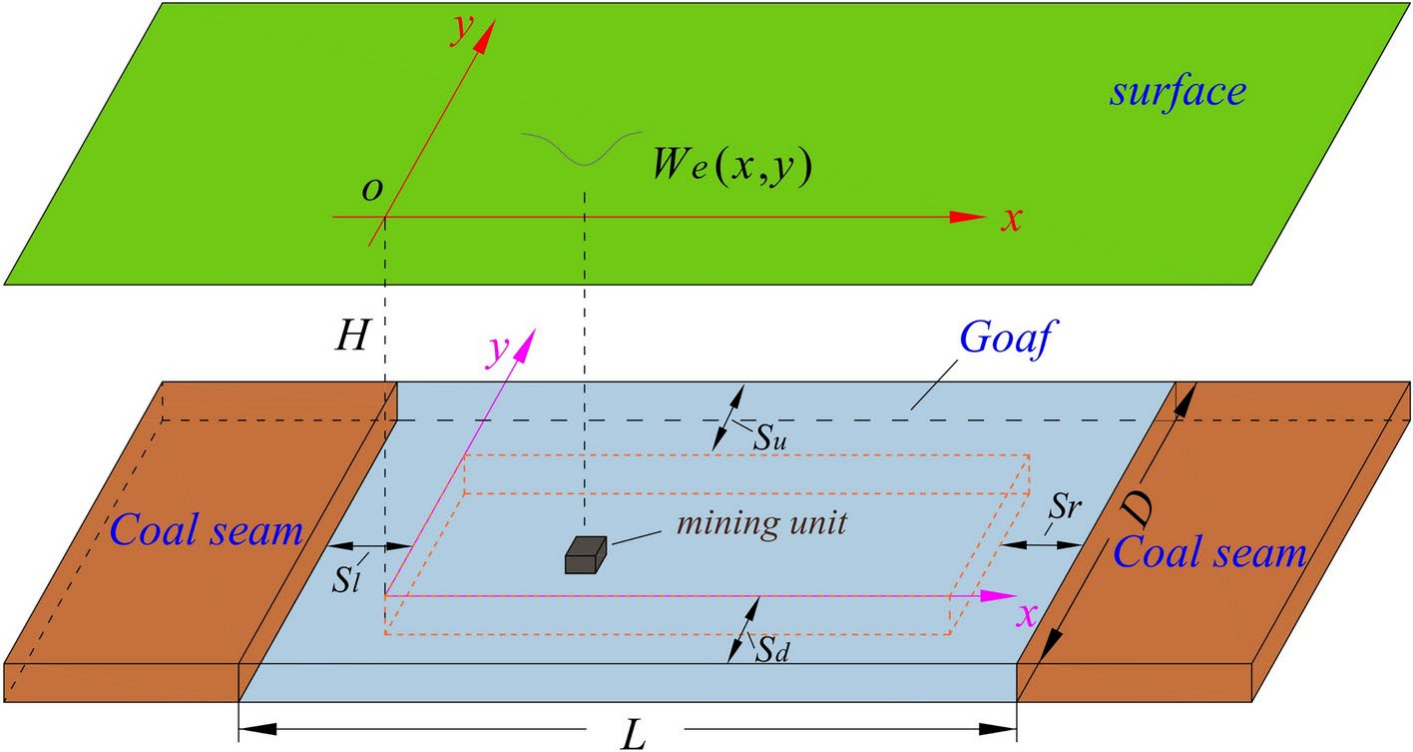
Model of surface subsidence caused by the unit working face mining.

As shown in Fig. 2, the formula for surface subsidence, from a three-dimensional perspective, caused by mining a small unit working face with center coordinates (*s*, *t*) is:

2 $$\begin{aligned} W_{e}(x,y)=w_{e}(x)\cdot w_{e}(y)=\frac{1}{r^{2}} e^{-\pi \frac{(x-s)^{2}+(y-t)^{2} }{r^{2}}}. \end{aligned}$$

Taking into account the effect of coal seam inclination, when the coal seam thickness is *m*, the strike length of the working face is *L*, and the dip length is *D*, the surface subsidence caused by the whole working face mining can be obtained by accumulating the subsidence resulting from all small units mining:

3 $$\begin{aligned} W(x,y)=\int \limits _{0}^{l} \int \limits _{0}^{d} W_{0}W_{e}(x,y) \,ds\,dt=\int \limits _{0}^{l} \int \limits _{0}^{d} \frac{W_{0}}{r^2}e^{-\pi \frac{(x-s)^2+(y-t+\Delta l)^2}{r^2}} \,ds\,dt, \end{aligned}$$

where *W*(*x*, *y*) is the subsidence value of the ground point (*x*, *y*), and (*s*, *t*) is the center coordinate of the small unit mining. $W_{0}$ is the maximum subsidence value of the surface. Its formula is $W_{0}=mqcos\alpha$, in which *m* is the mining thickness of the working face, *q* is the subsidence coefficient, and $\alpha$ represents the dip angle in the coal seam. $\Delta l=Hcot\theta$, in which $\theta$ is the maximum subsidence angle. $l=L-S_l-S_r$, $d=D-S_u-S_d$, *L* and *D* represent the strike length and the dip length of the working face, respectively. $S_{l}$, $S_{r}$, $S_{u}$, and $S_{d}$ represent the offset values of left, right, upper, and lower inflection points, respectively. Above is the subsidence calculation method in the PIM.

### Dynamic subsidence prediction model by PIM

**Fig. 3:**
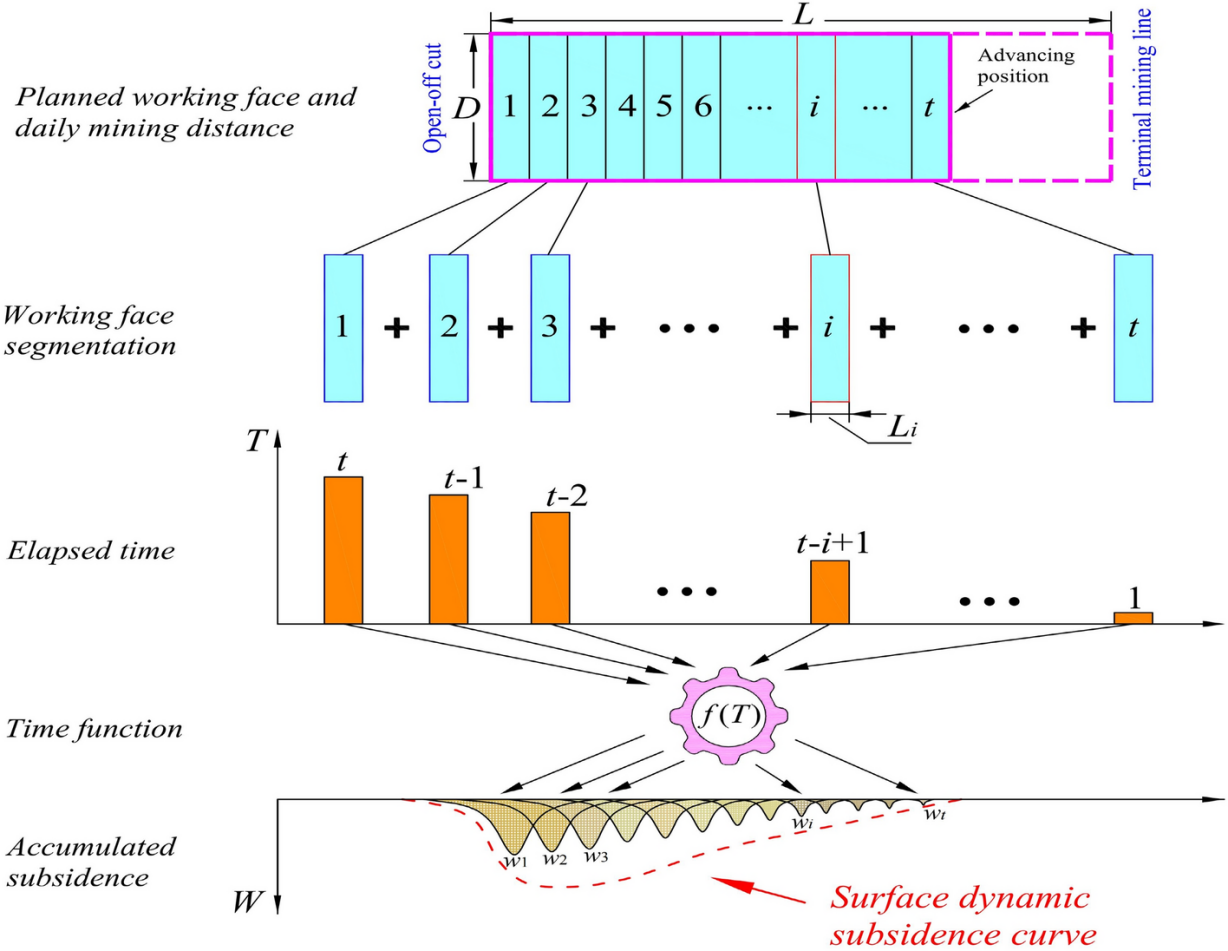
Process diagram of dynamic prediction model.

According to the PIM method, the ‘final’ subsidence value of surface points under the influence of mining can be determined. To calculate the dynamic subsidence value of surface points at a certain moment *t*, it is necessary to combine the actual daily advancing distance of underground mining with a time function to construct a mathematical model that establishes the relationship between the final subsidence and the dynamic subsidence at moment *t*. When a planned working face has been mined for *t* days, the impact time of the small working faces formed each day on the surface points is different. The surface subsidence impact from the small working face created on the first day of mining has lasted for *t* days, the impact from the second day has lasted for *t*-1 days, the impact from the third day has lasted for *t*-2 days, and so on. The mining impact on the $t_{th}$ day lasts just for 1 day. In the random media theory, the working faces formed each day can be separated, and the surface dynamic subsidence caused by each daily working face can be calculated individually. Finally, the surface dynamic subsidence on the *t* day can be obtained through accumulation, as shown in Fig. 3.

The basic process of dynamic subsidence prediction can be divided into the following calculation steps:

*Step 1* Based on the actual mining speed of the working face, let the starting time of the open-off cut position be equal to 0, and divide the working face according to the daily advancing distance to obtain *t* small unit working faces. The advancing length on the $i_{th}$ day is $L_{i}$. The final subsidence value $W_{0}(i,p)$ of surface point *p*(*x*, *y*) caused by the small working face *i*, which has dimensions of $D\times L_{i}$, can be calculated using formula (3).

*Step 2* Calculate the impact time of small working faces formed by daily mining on surface subsidence. On the $t_{th}$ day, the influence duration of the small working face formed on the $i_{th}$ day on surface subsidence is *T*= *t*- *i* + 1 days.

*Step 3* Using the time function *f*(*T*), the subsidence value *W*(*i*, *p*, *t*) caused by unit working face *i* at surface point *p* on the $t_{th}$ day can be calculated as:

4 $$\begin{aligned} W(i,p,t)=W_{0}(i,p)\cdot f(T). \end{aligned}$$

*Step 4* Accumulate the dynamic subsidence effects *W*(*i*, *p*, *t*) on ground point *p* from all mined unit working faces at time *t* to obtain the predicted dynamic subsidence *W*(*p*, *t*) at point *p* at time *t*.

5 $$\begin{aligned} W(p,t)=\sum _{i=1}^{t} W(i,p,t). \end{aligned}$$

Similarly, the movement and deformation metrics, such as dynamic inclination and dynamic curvatures, can be calculated at all points at time *t*.

## Time function and its parameter

From the principle of dynamic subsidence prediction based on the PIM method, it can be understood that the accuracy of both the PIM method and the time function determines the precision of dynamic subsidence prediction. Currently, under the premise of accurate PIM parameters, the prediction error for the final subsidence obtained using the PIM method can be controlled within 5%, which meets the accuracy requirements for most engineering^24^. Therefore, regarding the prediction of dynamic surface subsidence and its accuracy issues, the focus is primarily on the study of dynamic time function *f*(*T*).

### Classical time function and its parameter determination method

In recent years, many scholars have proposed various dynamic prediction time functions based on the dynamic subsidence process of surface points, such as the Knothe function^15^, Logistic function^16^, Weibull function^17^, Gonzalez function^25^, piecewise Knothe function^26^, normal time function^27^, and so on^18–21^. Among these time functions, the Knothe function is one of the most classical time functions, with advantages such as having fewer unknown parameters, clear physical meaning, and strong generalizability, it is helpful for us to conduct subsequent studies and propose a new prediction method. Therefore, this paper selects the classical Knothe function as the time function for dynamic subsidence prediction. This function is based on the assumption that the velocity of surface subsidence is proportional to the difference between the final subsidence value and the dynamic subsidence value at time *T*, namely:

6 $$\begin{aligned} \frac{dW(i,p,T)}{dt} =c\left( W_0(i,p)-W(i,p,T) \right) , \end{aligned}$$

where, *c* is the parameter of Knothe time function.

By solving this differential equation and incorporating the initial condition $W(i,p,T)=0$ when $T=0$, the expression for the instantaneous subsidence value of the surface point at time *T* is obtained as follows:

7 $$\begin{aligned} W(i,p,T)=W_0(i,p)\left( 1-e^{-cT} \right) . \end{aligned}$$

Therefore, for Knothe function, the expression of *f*(*T*) is as follows:

8 $$\begin{aligned} f(T)=1-e^{-cT}. \end{aligned}$$

The selection of parameter *c* is particularly important for the accuracy of surface dynamic subsidence prediction. The value of parameter *c* is related to the mining method of the working face and the properties of the overburden. Cui et al.^28^ believed that the value of *c* is related to the advancing speed of the underground working face based on a large amount of measured data, and provided a range for the parameter *c*. This was later developed by Wu et al.^29^, who proposed the classical calculation formula for *c*:

9 $$\begin{aligned} c\approx 2v\tan \beta /H_{0}. \end{aligned}$$

In the formula, *v* represents the average advancing speed of the working face, and $H_{0}$ is the average mining depth.

### Adaptive calculation method for time function parameter

#### Inadaptability of classical time function

**Fig. 4:**
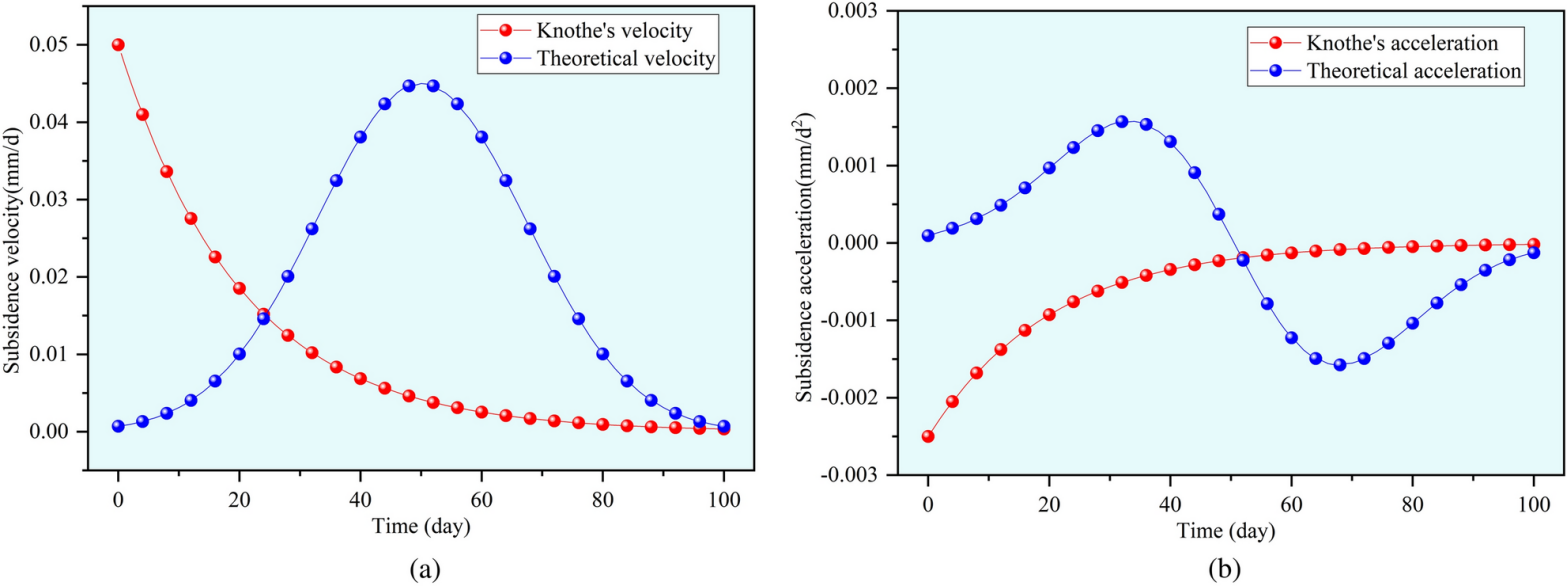
Comparison of characteristics of surface subsidence caused by underground mining.

Liu^22^ studied and concluded that the Knothe time function cannot reflect the dynamic subsidence process of surface points influenced by underground mining activities. The main reason is that by differentiating the Knothe time function, a curve describing the subsidence velocity and subsidence acceleration of surface points can be obtained, as shown in Fig. 4. This reflects a subsidence process characterized by decreasing acceleration, which differs significantly from the actual subsidence process caused by underground mining, where surface points first accelerate and then decelerate. This discrepancy leads to the issue of the classical Knothe time function’s inadaptability.

**Fig. 5:**
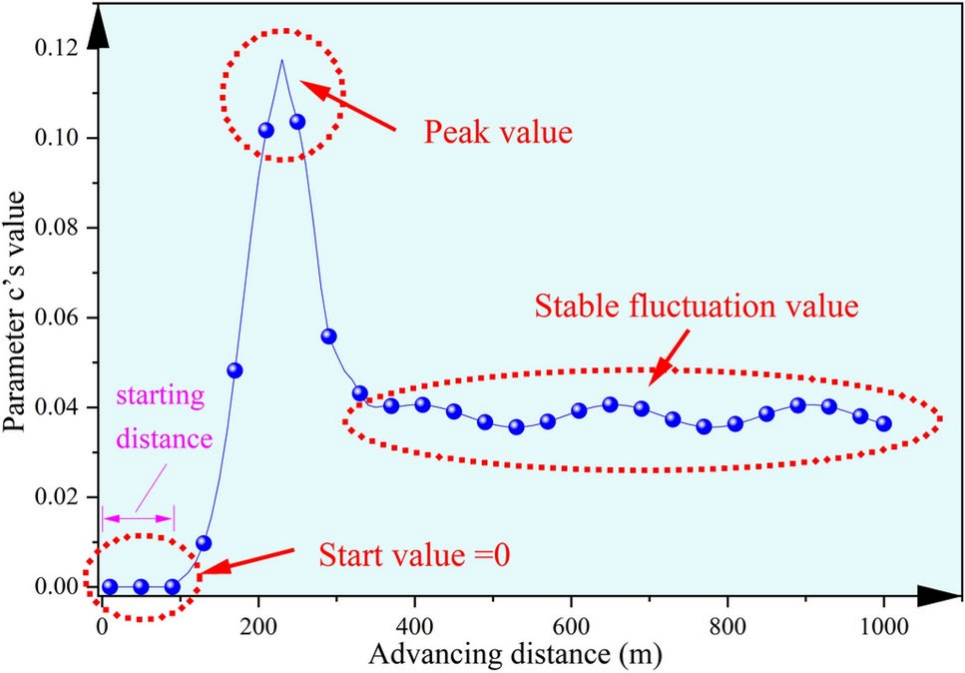
Theoretical dynamic variation curve of parameter c with advancing distance.

On the other hand, from a physical perspective, the parameter *c* in the Knothe time function actually represents the delay effect of subsidence caused by underground mining as it propagates through the overburden^30^. This delay effect is influenced by the position of underground advancing and the state of the overburden structure. Theoretically, when the underground working face starts mining until it reaches the starting distance, the optimal parameter value *c* is 0. As the working face advances beyond the starting distance, surface subsidence begins; however, at this point, the structural integrity of the key stratum is still intact and can support the weight of its own and overburden. Therefore, the delay effect is maximized, and the value of *c* peaks. Once further mining leads to the failure of the key stratum, the integrity of the overburden is compromised, significantly reducing its load-bearing capacity. This, combined with the release of previously accumulated potential energy, results in rapid surface subsidence. At this stage, the delay effect quickly diminishes, and the value of *c* rapidly decreases. As mining continues and the overburden structure experiences periodic deformation and failure, the delay effect on surface subsidence oscillates periodically, causing the value of *c* to fluctuate around a certain fixed value. Therefore, the theoretical curve of parameter *c* as it varies with the underground advancing length should be as shown in Fig. 5.

Therefore, the value of parameter *c* is constantly changing, making it difficult to determine a specific value directly. The inaccuracies in the parameter *c* provided by classical formulas are also a significant reason for the low accuracy in predictions of surface dynamic subsidence.

#### Principles of the adaptive model

The aforementioned analysis results indicate that the classical parameter *c* calculation model finds it difficult to account for the differences in the delay effects caused by the dynamic changes in the overburden structural state during underground mining activities, thereby affecting the accuracy of surface dynamic subsidence predictions. Therefore, an attempt is made to develop a variable parameter *c* to improve the adaptability of the Knothe function in surface dynamic subsidence prediction. Considering the need to ensure the safety of some large and important structures, it is generally necessary to conduct high-frequency deformation monitoring during underground mining activities. Therefore, it is entirely possible to use the existing historical measured data for dynamic feedback correction and extrapolative prediction of the parameter *c* value. This method makes full use of underground mining information and historical monitoring data of surface subsidence, making the subsidence prediction results more accurate and reliable. Based on this, this paper proposes an adaptive dynamic subsidence prediction model aided by measured data. Taking the prediction of dynamic subsidence at the surface for the advancing to position $d_{N+1}$ as an example, if historical monitoring data from *N* periods have been obtained before reaching $d_{N+1}$, the steps for the prediction are as follows.

*Step 1* First, backtrack *M*($M<N$) periods (i.e.,(*N*, *N*-1,..., *N*-*M*+1)) of measured data (i.e.,($S_{N}$, $S_{N-1}$, ..., $S_{N-M+1}$)) from the predicted time to obtain a sequence of the optimal parameter *c* values for each period (i.e.,($c_{N}$, $c_{N-1}$, ..., $c_{N-M+1}$)).

*Step 2* Based on the corresponding underground advancing position for the nearest *M* monitoring periods ($d_{N-M+1}$,..., $d_{N-1}$,$d_{N}$) and the corresponding sequence of optimal fitting parameter *c* values ($c_{N-M+1}$,...,$c_{N-1}$,$c_{N}$), extrapolate the parameter $c_{N+1}$ value when advancing to position $d_{N+1}$.

*Step 3* Combine the underground mining information at position $d_{N+1}$ with the parameter $c_{N+1}$, and substitute them back into the dynamic prediction model to estimate the surface dynamic subsidence $S_{N+1}$ when mining reaches position $d_{N+1}$.

*Step 4* As the underground mining activities progress and measured data of surface subsidence are continuously collected, repeat step (1) to (3) to predict the surface dynamic subsidence values at different advancing positions.

**Fig. 6:**
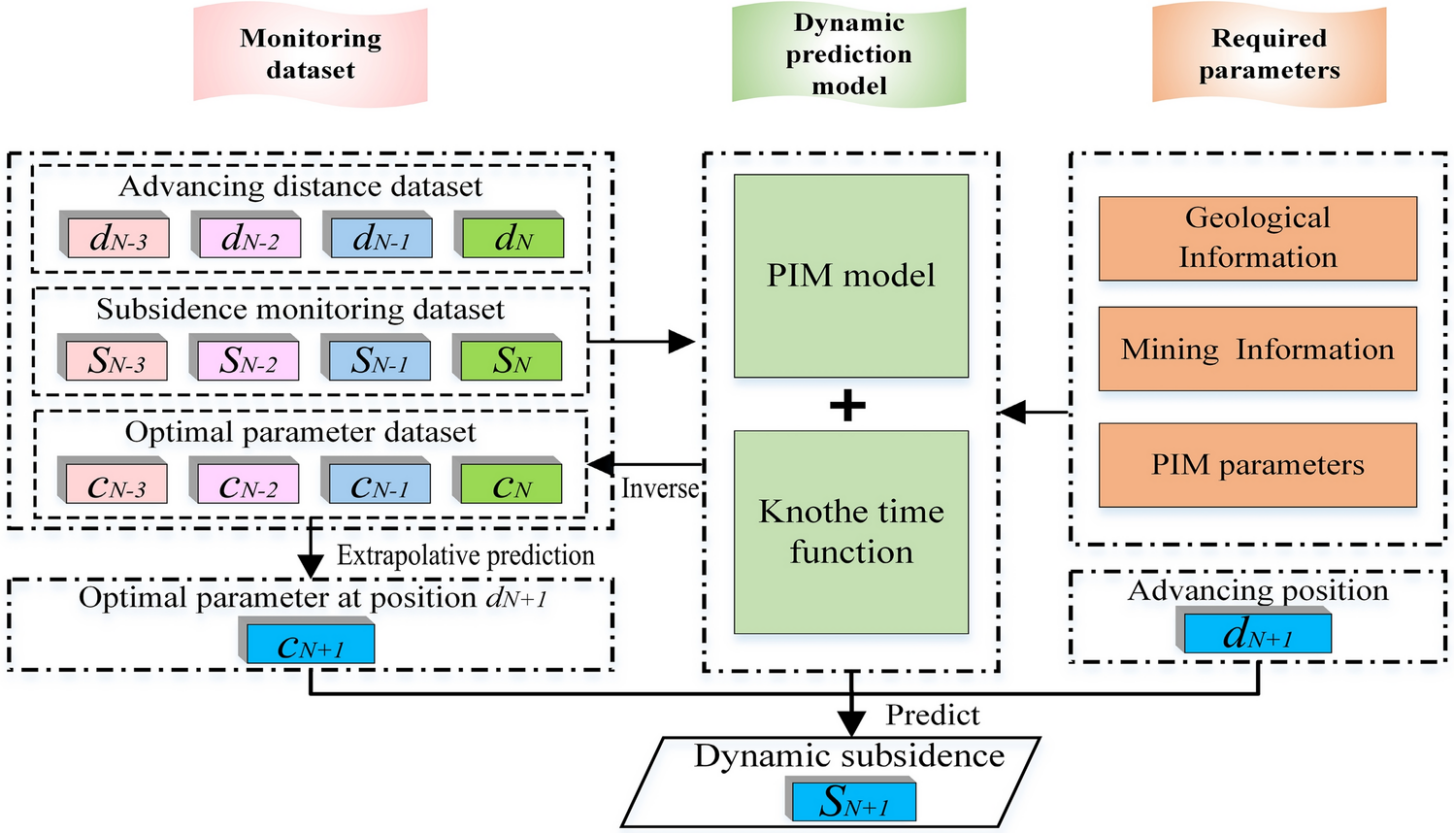
Flowchart of the adaptive dynamic surface subsidence prediction model aided by measured data.

This model requires the use of an extrapolation model to predict the parameter $c_{N+1}$ value for the next advancing position $d_{N+1}$. Common extrapolation prediction models include Kalman filtering, neural networks, Bayesian approaches, and so on. Most of these extrapolation methods rely on a large amount of prior data and prior information. However, in traditional surface monitoring tasks with low monitoring frequency and large temporal spans, it is challenging to have sufficient sample data for training and extrapolation prediction, which may lead to unstable prediction results or even make extrapolation prediction impossible. The gray prediction model is characterized by requiring few data points, having high prediction accuracy, and not needing prior information^31^. Considering the influence of random disturbing factors on the gray prediction model, this study adopts the approach of the Metabolism Model^32^. It utilizes recently measured data to discard outdated early data, thereby extrapolating parameter *c* value that better aligns with recent observational features. This time series extrapolation model requires at least 4 periods of historical monitoring data ($M\ge 4$), meaning that for each prediction, the calculations and parameter extrapolation are based on the measured data from the previous *M* periods. Taking $M=4$ as an example, the algorithm flowchart of the adaptive model is shown in Fig. 6.

The model involves a large amount of data and parameters, making data management relatively complex and resulting in a significant computational workload, with a computational complexity of $O(N_P*T)$ (where $N_P$ is the number of predicted points and *T* is the prediction time). Therefore, we divided the prediction model system into three modules using a computer program: monitoring data acquisition and processing, probability integral methods for dynamic subsidence prediction, and geological mining parameters management. This program supports data sharing and function calls, significantly reducing the workload and improving prediction efficiency. In this study, we used the Visual Basic programming language to write the program code. Taking a local computer with a Windows 10 and 32 GB RAM as an example, dynamic predictions for 10,000 ($N_P$=10,000) surface points on day 10 ($T=10$) require approximately 7s, indicating that the program’s efficiency can meet current engineering prediction needs. Furthermore, future improvements may include optimizing the code and data management approaches, as well as adopting parallel computing methods to enhance the program’s efficiency and practicality in real-world applications.

## Case study

### Study area overview

**Fig. 7:**
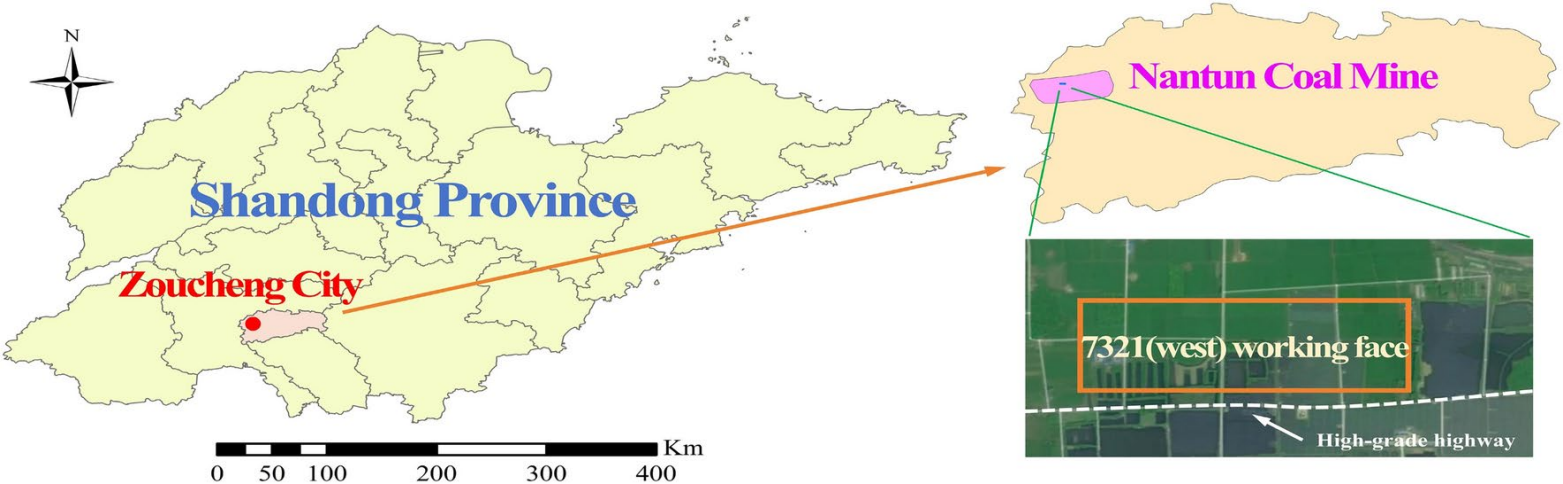
Geographic position of the study area (The map was generated by the authors with the help of ArcGIS 10.6 (https://support.esri.com/en/download/7583) and does not require any permission from anywhere).

The Nantun Coal Mine, operated by Yanzhou Coal Mining Company Limited, is located in Zoucheng City, Shandong province, as shown in Fig. 7. The minefield has an east-west length of 10.5 $\textrm{km}$, a north-south width of 5.2 $\textrm{km}$, and an area of 54.6 $\textrm{km}^2$. The regional strata consist of the Ordovician, Carboniferous, Permian, Jurassic, and Quaternary systems.

**Fig. 8:**
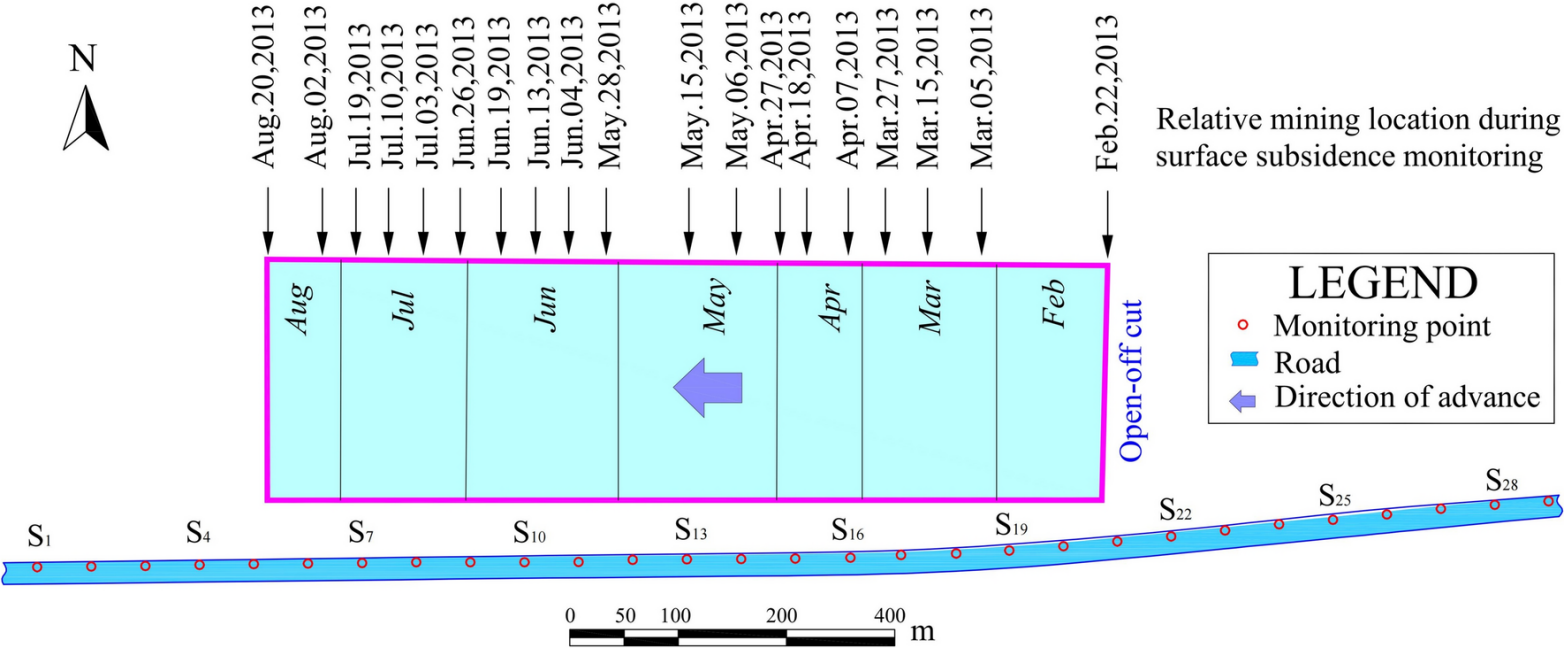
Relative position of 7321 (west) working face and highway.

**Fig. 9:**
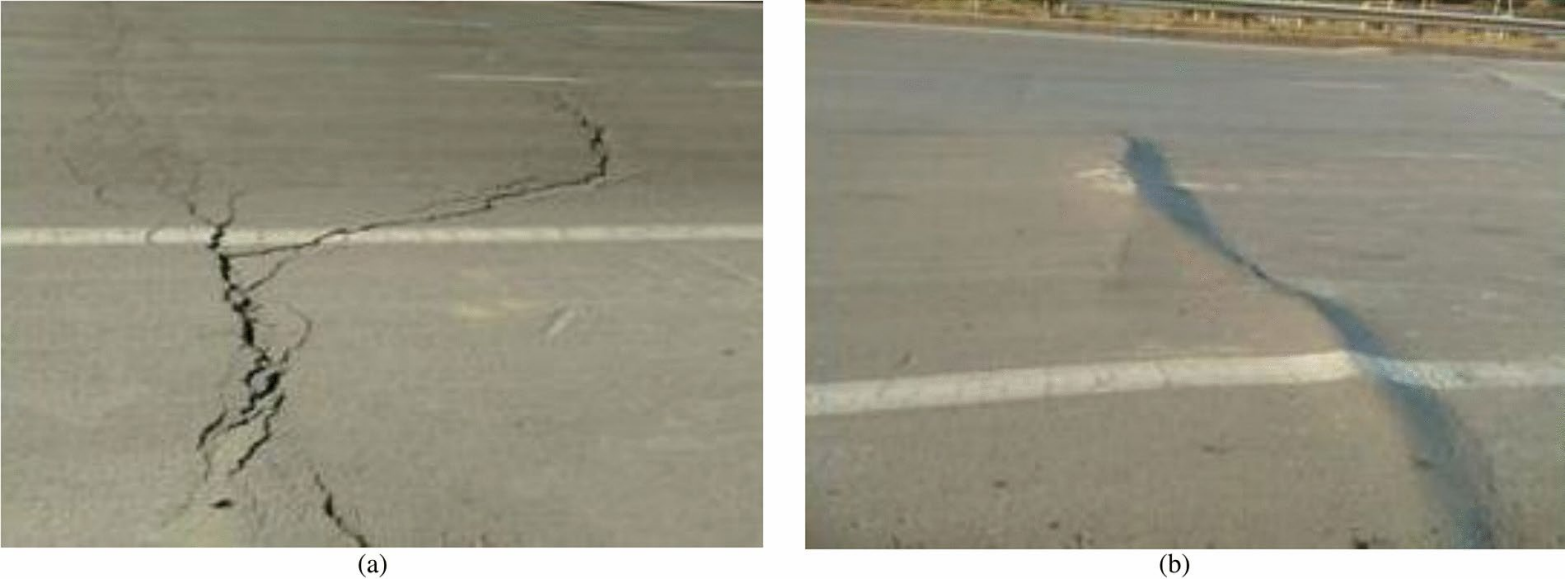
Highway damage conditions. (**a**) Cracks. (**b**) Bumps.

The No. 7321 (west) working face in Nantun Coal Mine is the experimental working face, and a high-grade highway from Zoucheng to Jining passes directly above it. Figure 8 shows the relative positions of the working face and the highway. The underground mining began in February 22, 2013 and concluded on August 18, 2013. Mining proceeded from east to west. The average thickness of the coal seam is 3.4 m, with a dip angle of $6^{\circ }$. The strike length of the working face is 776.3 m, and the dip length is 214.7 m. The average mining depth is 500 m, and the area of the working face is approximately 166,700 $\text {m}^{2}$. The average advance velocity is 4.38 m/d. As the 7321 (west) working face advances from east to west, the highway suffers significant damage, such as cracks and bumps, which seriously disrupt safe vehicular operations (as shown in Fig. 9).

**Fig. 10:**
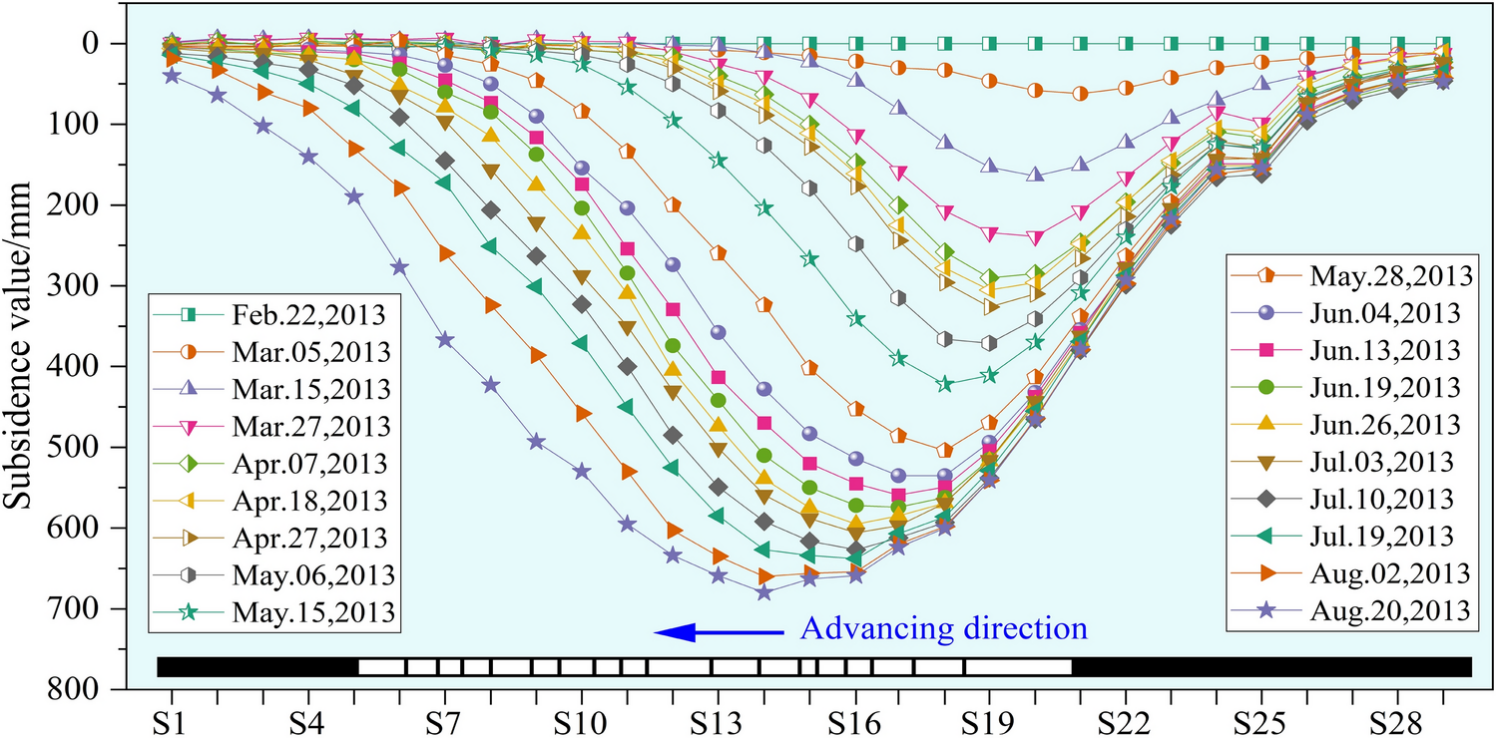
Dynamic change process of highway subsidence.

To provide timely predictions and maintenance for the damages to the highway, surface movement observation stations have been installed on the highway pavement, and the leveling method is used for monitoring surface subsidence. The observation period was from February 2013 to August 2013, during which a total of 19 measurements were conducted, collected a large amount of data on highway movement and deformation. The subsidence curves of the highway surface during every period are shown in Fig. 10.

It can be observed that the subsidence of the right-side road exhibited significant variability in the initial periods but gradually stabilized thereafter. Meanwhile, as the underground working face progressed, the subsidence impact on the left-side road gradually expanded, with the maximum subsidence value reaching 680 mm. Among the observation points, only point S25 displayed some anomalies in its data, and other points were relatively normal, aligning with the general morphology of the subsidence basin.

### Comparison of dynamic surface subsidence prediction models

**Fig. 11:**
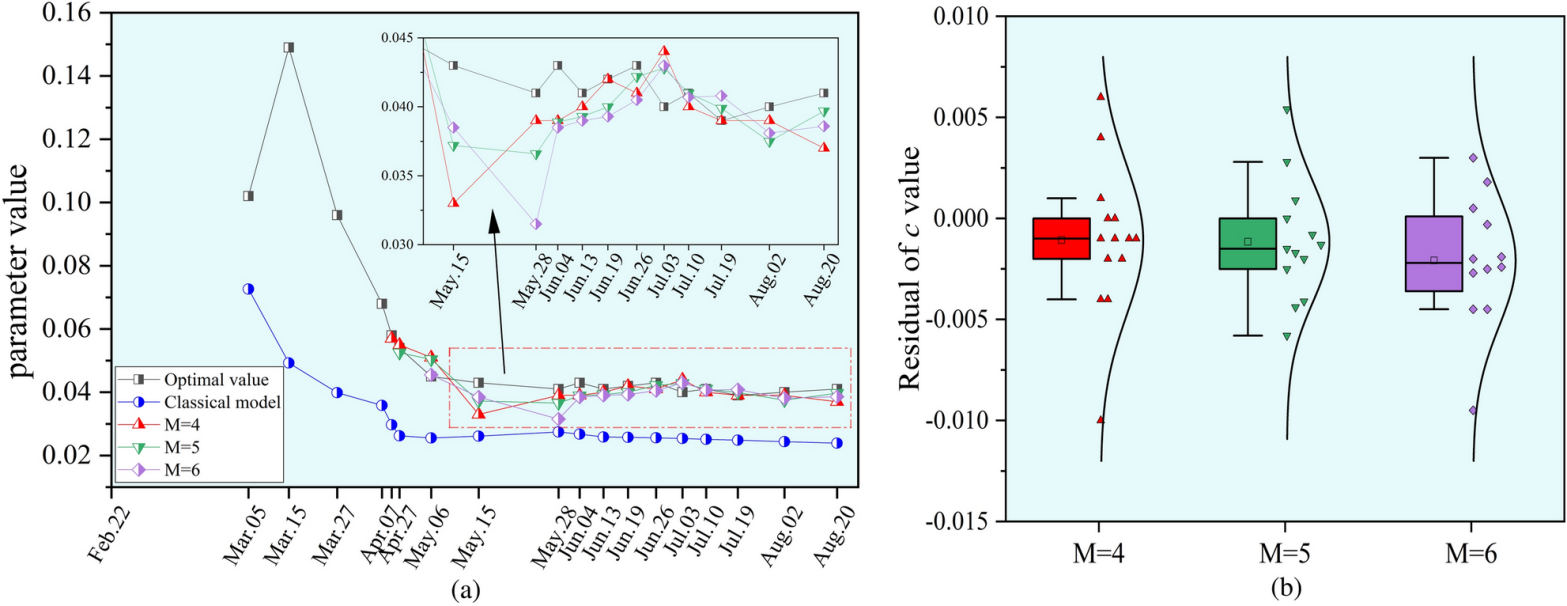
Comparison of the values of the parameter *c* calculated by each model for each period. The curve chart of parameter *c* is shown in (**a**). The relative error distribution of parameter *c* is shown in (**b**).

To provide reference data for the selection of road maintenance methods and the scheduling of engineering activities by highway departments, it is essential to conduct timely predictions and assessments of road surface subsidence and damage levels during the underground mining process. The dynamic prediction method for surface subsidence is employed to carry out dynamic forecasting of highway subsidence. First, the classical model parameter *c* is calculated according to Formula (9) using the historical average advancing speed at the predicted time. The optimal *c* value is derived from the inversion of the measured subsidence data for each period. Subsequently, the optimal *c* value from the previous *M* periods is used to extrapolate the parameter *c* value of the adaptive model beginning from the fifth period. We compared the prediction results of parameter *c* for the values of *M* = 4, 5, and 6. The comparison results are presented in Fig. 11.

The results indicate that different values of parameter *M* result in the predicted parameter *c* fluctuating around the optimal value, with slight differences in the optimal extrapolated parameter *c*. Overall, using a smaller *M* value maintains high sensitivity to recently added data, allowing for quicker adaptation to recent data changes, and the predicted surface dynamic subsidence is more aligned with recently measured data. Furthermore, a smaller *M* value enables the practical application of this method in early engineering phases, which is significant for early dynamic subsidence predictions. So, we set $M=4$, and the calculation results of parameter *c* for each period are shown in Table 1.

**Table 1:** Accuracy comparison of parameter *c* for each period.

Monitoring date	Advancing distance/m	Optimal value	Classical model		Adaptive model	
			Calculated value	Relative error/%	Calculated value	Relative error/%
Mar.05	156	0.102	0.073	28.8	–	–
Mar.15	202	0.149	0.049	66.9	–	–
Mar.27	257	0.096	0.040	58.5	–	–
Apr.07	308	0.068	0.036	47.3	–	–
Apr.18	319	0.058	0.030	48.8	0.057	1.7
Apr.27	328	0.054	0.026	51.4	0.055	1.9
May.06	364	0.045	0.026	43.3	0.051	13.3
May.15	418	0.043	0.026	39.3	0.033	23.3
May.28	509	0.041	0.027	33.1	0.039	4.9
Jun.04	533	0.043	0.027	37.8	0.039	9.3
Jun.13	560	0.041	0.026	37.0	0.040	2.4
Jun.19	588	0.042	0.026	38.7	0.042	0.0
Jun.26	620	0.043	0.026	40.5	0.041	4.7
Jul.03	650	0.040	0.025	36.5	0.044	10.0
Jul.10	677	0.041	0.025	38.7	0.040	2.4
Jul.19	713	0.039	0.025	36.3	0.039	0.0
Aug.02	766	0.040	0.024	39.1	0.039	2.5
Aug.20	826	0.041	0.024	41.7	0.037	9.8

**Fig. 12:**
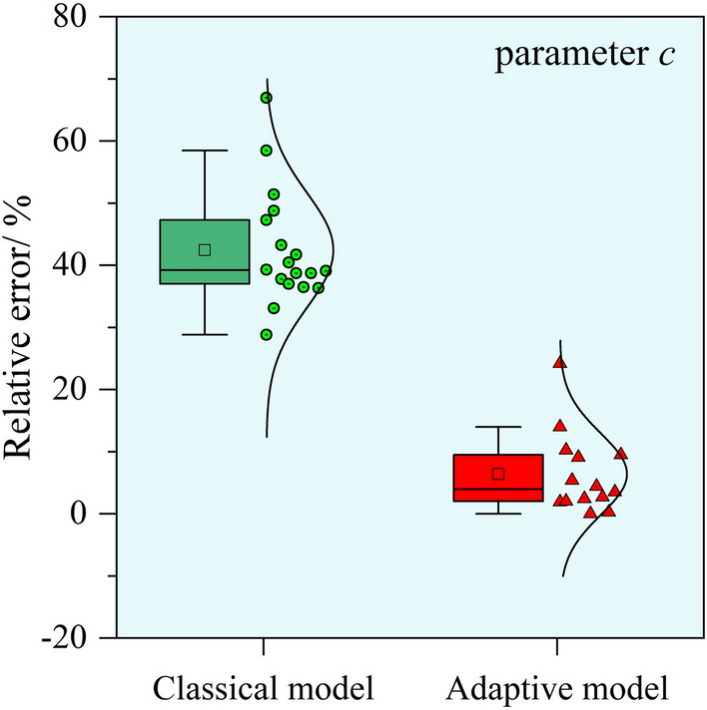
The distribution of the parameter *c*.

Table 1 shows that the value of *c* is influenced by the underground advancing distance, resulting in distinctly different optimal values of parameter *c* at different periods. The average relative error of the parameter *c* calculated using the classical model is 40.1%. In contrast, when employing the adaptive model proposed in this study for the extrapolation calculation of parameter *c*, the prediction errors for most periods are within 10%, with larger errors occurring only for the periods when the advancing distance reached 364 m and 418 m. We plotted the box plots of the relative error distribution for the extrapolated predicted values of parameter *c*, as shown in Fig. 12. It is evident that the prediction accuracy of parameter *c* in the adaptive model has significantly improved.

**Fig. 13:**
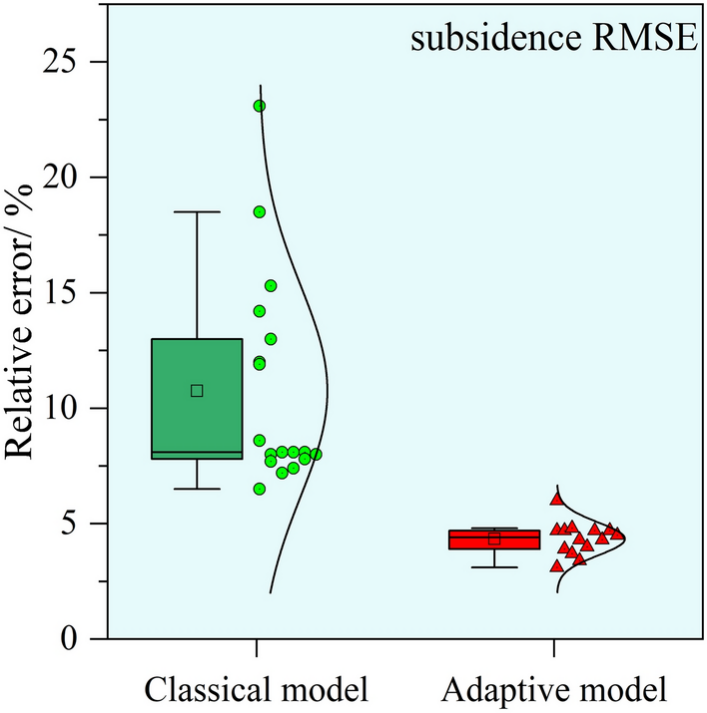
The distribution of RMSE.

Using the calculated values of parameter *c* for each period, the predictive performance of the classical model and the adaptive model on dynamic surface subsidence was compared, as presented in Table 2. We plotted the box plots of the relative error distribution of the subsidence fitting RMSE, as shown in Fig. 13.

**Table 2:** Comparison of fitting effects between predicted and measured subsidence.

Monitoring date	Advancing distance/m	Classical model		Adaptive model	
		RMSE/mm	Relative error/%	RMSE/mm	Relative error/%
Mar.05	156	7.4	12.0	–	–
Mar.15	202	37.8	23.1	–	–
Mar.27	257	44.3	18.5	–	–
Apr.07	308	37.6	13.0	–	–
Apr.18	319	43.2	14.2	14.2	4.7
Apr.27	328	50.0	15.3	15.3	4.7
May.06	364	44.2	11.9	17.7	4.8
May.15	418	36.2	8.6	25.5	6.0
May.28	509	32.6	6.5	15.8	3.1
Jun.04	533	42.7	8.0	20.7	3.9
Jun.13	560	43.1	7.7	20.5	3.7
Jun.19	588	46.7	8.1	24.6	4.3
Jun.26	620	48.0	8.1	23.7	4.0
Jul.03	650	43.5	7.2	20.7	3.4
Jul.10	677	50.8	8.1	29.3	4.7
Jul.19	713	47.4	7.4	27.4	4.3
Aug.02	766	52.9	8.0	31.0	4.7
Aug.20	826	52.7	7.8	30.4	4.5

Starting from the Apr.18 period, the classical model’s maximum RMSE for fitting dynamic subsidence is 52.9 mm (on Aug.02), with a maximum relative RMSE of 15.3% (on Apr.27) and an average relative RMSE of 9.1%. The adaptive model has a maximum RMSE of 30.4 mm (on Aug.20), a maximum relative RMSE of 6.0% (on May.15), and an average relative RMSE of 4.3%. The t-test results of the residuals from the subsidence predictions of the two models yield *p* $< 0.05$. It is evident in Fig. 13 that the predictive accuracy of subsidence using the adaptive model has significantly improved compared to the classical model.

**Fig. 14:**
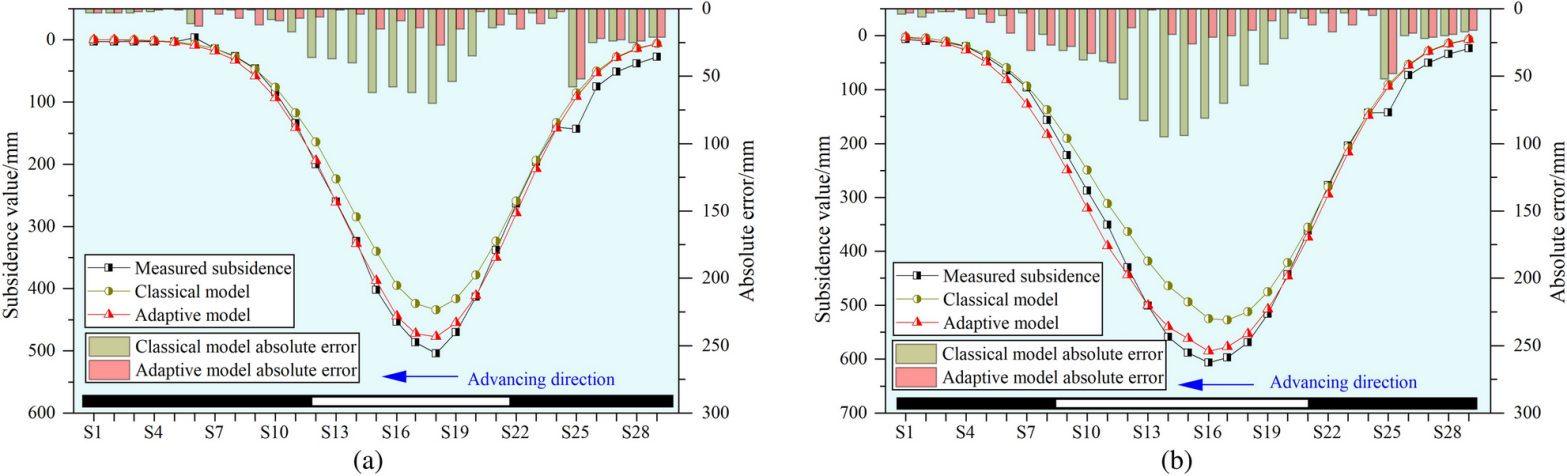
Comparison of predicted results of road dynamic subsidence. The date May.28 is shown in (**a**). The date Jul.03 is shown in (**b**).

The comparison of predicted and measured subsidence values for two monitoring periods (May.28 and Jul.03) is presented in Fig. 14.

Through comparative analysis, it can be concluded that on May.28, the fitting effects of the two methods are largely similar at points S21 to S29. However, the classical model demonstrates poor fitting performance at points S10 to S21, primarily due to the smaller computed value of the time function parameter *c* in the classical model (0.027 < 0.039). Because a smaller parameter *c* value results in a longer time for the surface points to reach the final subsidence, the subsidence curve exhibits a noticeable right-skewed distribution effect. On Jul.03, in addition to the pronounced right-skewed distribution resulting from the smaller parameter *c* value calculated by the classical model, the computed value of the time function parameter *c* for the adaptive model (0.044 > 0.040) is slightly larger, which causes the right-skewed effect of the subsidence curve predicted by the adaptive model to be somewhat insufficient. Overall, on May.28 and Jul.03, the RMSE values for subsidence predictions from the adaptive model were 15.8 mm and 20.7 mm, respectively, significantly lower than the classical model’s RMSE values of 32.6 mm and 43.5 mm. These results indicate that the adaptive model exhibits a substantial improvement in subsidence prediction accuracy compared to the classical model.

**Fig. 15:**
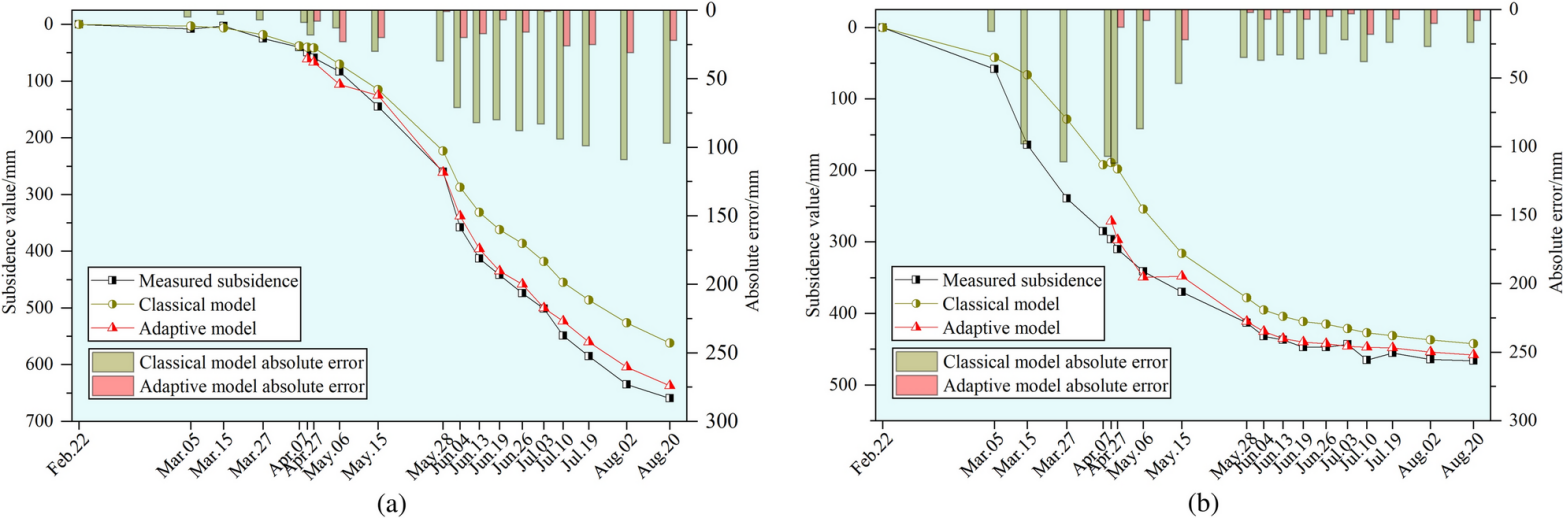
Comparison of dynamic subsidence prediction results at observation points over time. The point S13 is shown in (**a**). The point S20 is shown in (**b**).

Select point S13, located in the center of the mined-out area, and point S20, situated near the boundary of the mined-out area, to predict pavement subsidence using both models for all periods and compare the results, as shown in Fig. 15.

As illustrated in Fig. 15, the parameter *c* value predicted by the classical model is generally underestimated, leading to smaller predicted dynamic subsidence values. Meanwhile, the parameter *c* value from the adaptive model on May.15 exhibited slight errors, resulting in anomalies in the predicted dynamic subsidence values. Overall, the maximum residuals of the predicted results for points S13 and S20 in the classical model are 109 mm and 112 mm, corresponding to relative errors of 16.5% and 24% with respect to the final subsidence values. In comparison, the maximum residuals for the subsidence results of points S13 and S20 predicted by the adaptive model are 31 mm and 32 mm, which yield relative errors of 4.7% and 6.9% relative to the final subsidence values. It is evident that the predictive accuracy of the adaptive model is significantly higher than that of the classical model, regardless of whether the surface points are located in the center or along the edge of the mined-out area. The error in the dynamic subsidence predictions for individual points remains within 7%.

**Fig. 16:**
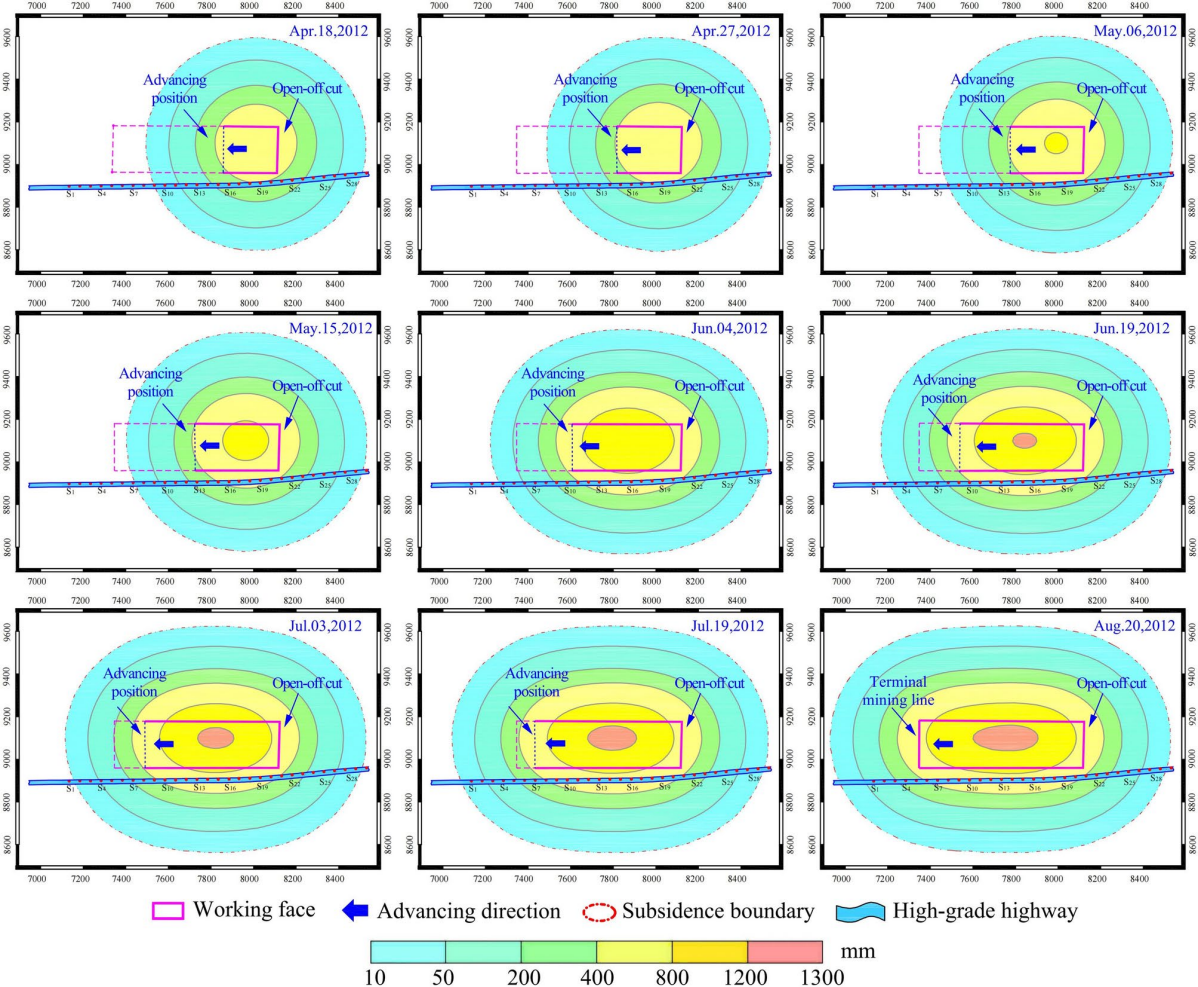
Dynamic changes of the predicted ground subsidence contour map.

Using the adaptive dynamic subsidence prediction model presented in this study, the dynamic changes in the contour map of surface subsidence during the underground mining process at working face 7321 (west) were predicted, as shown in Fig. 16.

It can be seen from Fig. 16 that as the underground working face progresses continuously to the left, the extent of the surface subsidence basin also extends to the left. At the same time, the maximum subsidence value gradually increases, ultimately reaching approximately 1300 mm. The highway is situated within the subsidence-influence area and is significantly affected by mining activities. On the right side, the influence extends to the vicinity of point S29, while the left boundary of the influence zone continuously changes with the underground mining activities, ultimately reaching a point 160 m to the left of point S1. The subsidence basin in the mining area is not completely symmetrical; specifically, on Aug.20, it displays an ‘egg-shaped’ contour of subsidence, with the left side representing the small end of the egg and the right side representing the larger end. This reflects a delay in subsidence on the left side of the mining area as of Aug.20, indicating that subsidence values will continue to increase to some extent, ultimately leading to the formation of a symmetrically shaped subsidence basin.

## Discussion

The adaptive model can effectively enhance the prediction accuracy of surface dynamic subsidence, primarily relying on the reliability of the subsidence prediction model and its parameters. The PIM model has been validated through decades of engineering practice and has been applied under various geological mining conditions, reflecting the reliability of the PIM model. However, the PIM parameters still require continuous monitoring and the accumulation of historical monitoring data to analyze the subsidence characteristics under specific geological mining conditions to determine reliable PIM parameters.

The core of the adaptive model of subsidence dynamic prediction lies in the continuous adjustment of the parameter *c* of the Knothe function using the measured surface subsidence data. This adjustment method of parameter *c* takes into account the combined effects of multiple factors, such as underground advancing speed and changes in overburden structure, thereby ensuring the stability of the key parameter *c* and preventing it from deviating too far, which could lead to inaccurate surface dynamic subsidence prediction results. Therefore, compared to the classical model that uses a fixed parameter *c* calculation formula, the adaptive method proposed in this study demonstrates significant reliability and flexibility.

The parameter *c* value varies continuously with changes in underground mining, making time series predictions using extrapolation methods possible. The metabolism model exhibits strong sensitivity to recent data, and selecting a smaller *M* value allows for accurate capture of recent data trends, thereby enabling precise prediction of the parameter *c* value in the next period. With the advancement of surface monitoring technologies and sensors, high-frequency monitoring methods can be employed in the future to gather substantial data, allowing us to further explore other extrapolation prediction models or setting a new *M* value, while simultaneously addressing the sensitivity and stability requirements of the parameter *c* prediction results.

In the protection engineering of important buildings, surface displacement and deformation monitoring are typically conducted. This provides the fundamental source of measured data for this model. Using the accurate surface subsidence data predicted by this model, a comprehensive analysis of the cooperative subsidence patterns between the buildings and the ground can be performed. By employing methods such as building reinforcement and underground grouting, real-time and accurate assessments of the engineering quantities and grouting volumes can be made, ensuring an accurate balance between the maintenance influence and the mining-influenced subsidence. This aims to maximize the control of ground subsidence and achieve the ultimate goal of protecting the safety of important buildings.

With the development of real-time monitoring technology and data transmission technology, monitoring data will become more diversified. It may be necessary to integrate heterogeneous data from multiple sources, such as remote sensing monitoring data and geological borehole displacement data, for fusion analysis to achieve comprehensive subsidence predictions from the ground to the rock layers. This requires the adoption of more comprehensive data processing, fusion, and analysis methods, combined with technologies such as network integration, parallel computing, and distributed computing to optimize algorithm processing efficiency, thereby further enhancing the effectiveness and reliability of the prediction results.

## Conclusions

The increasing prevalence of coal mining activities beneath large structures necessitates high-precision dynamic deformation predictions of the surface to develop effective maintenance and remediation strategies for damage caused by mining activities. The PIM method based on influence function theory has gained widespread application due to its ability to account for variations in underground mining activities while requiring fewer parameters. This study addresses the issue of the unsuitability of the PIM method combined with the classical Knothe time function model in predicting surface dynamic subsidence in mining areas. An adaptive dynamic subsidence prediction model aided by measured data was proposed and applied in engineering practice. The main research conclusions are as follows:

(1) This study analyzes the issues and underlying causes associated with the parameter calculation method for the classical Knothe function. It concludes that the classical formula does not account for the effects of underground advancing distance and the dynamic changes in overburden structures on the parameter *c*, resulting in decreased accuracy in dynamic subsidence predictions. Furthermore, the study elucidates the intrinsic significance of parameter *c* and the fundamental shape of its dynamic variation curve throughout the underground advancing process.

(2) An adaptive dynamic subsidence prediction model aided by measured data is proposed. This model utilizes measured subsidence data to invert and obtain a sequence of optimal parameter *c* values for each previous period. A metabolic model is then employed to extrapolate the optimal *c* value for the upcoming predicted period, thereby enhancing the accuracy of dynamic predictions.

(3) By analyzing the case of coal mining beneath a high-grade highway at the Nantun Coal Mine, this study conducts a comparative analysis of the application effectiveness of the classical dynamic subsidence prediction model and the adaptive dynamic subsidence prediction model proposed herein. The results indicate that the maximum relative RMSE for the classical model at each period is 15.3%, with an average relative RMSE of 9.1%. In contrast, the adaptive model achieves maximum and average relative RMSE of 6.0% and 4.3%, respectively, demonstrating a significant improvement in predictive accuracy.

(4) This model is applicable within a specific context. Given that it relies on historical monitoring data of surface subsidence, high-frequency monitoring of the ground surface is essential for the timely acquisition of subsidence data that can be integrated into the model. Notably, during the early stages of mining activities, when the parameter *c* exhibits rapid changes, increasing the monitoring frequency can enhance the accuracy of subsidence predictions. In projects involving protective engineering measures for critical surface structures, high-frequency subsidence monitoring and short-term dynamic predictions are typically required; therefore, this model is primarily tailored for such engineering applications.

## Supplementary Information

Supplementary Information 1.

Supplementary Information 2.

## Data Availability

All data generated or analysed during this study are included in its supplementary information files and available from the corresponding author at reasonable request.

## References

[1] The Energy Institute. *2024 Statistical Review of World Energy*. https://www.energyinst.org/statistical-review/home (2024).

[2] Williams, S., Bock, Y. & Fang, P. Integrated satellite interferometry: Tropospheric noise, gps estimates and implications for interferometric synthetic aperture radar products. *J. Geophys. Res.* **103**, 27051–27067. 10.1029/98JB02794 (1998).

[3] Bell, F. G., Stacey, T. R. & Genske, D. D. Mining subsidence and its effect on the environment: Some differing examples. *Environ. Geol.* **40**, 135–152. 10.1007/s002540000140 (2000).

[4] Doyle, P., Barton, P., Rosenbaum, M. S., Vandewalle, J. & Jacobs, K. Geo-environmental implications of military mining in Flanders, Belgium, 1914–1918. *Environ. Geol.* **43**, 57–71. 10.1007/s00254-002-0642-8 (2002).

[5] Kraatz, W. M. *Mining Damage and Its Protection* (Coal Industry Press, 1984).

[6] Bell, F. G. & Genske, D. D. The influence of subsidence attributable to coal mining on the environment, development and restoration: Some examples from western europe and south africa. *Environ. Eng. Geosci.* **7**, 81–99. 10.2113/gseegeosci.7.1.81 (2001).

[7] Cui, X. M., Che, Y. H., Zhao, Y. L., Li, P. X. & Bai, Z. H. Further discussion on mining deformation and building damage classification. *J. China Coal Soc.* **46**, 145–153 (2021).

[8] Wang, M., Lu, Z. X., Wan, W. & Zhao, Y. L. A calibration framework for the microparameters of the dem model using the improved pso algorithm. *Adv. Powder Technol.* **32**, 358–369. 10.1016/j.apt.2020.12.015 (2021).

[9] Wang, M. & Wan, W. A new empirical formula for evaluating uniaxial compressive strength using the Schmidt hammer test. *Int. J. Rock Mech. Min. Sci.* **123**, 104094. 10.1016/j.ijrmms.2019.104094 (2019).

[10] Wang, M., Wan, W. & Zhao, Y. Prediction of the uniaxial compressive strength of rocks from simple index tests using a random forest predictive model. *Comptes Rendus. Mécanique* **348**, 3–32. 10.5802/crmeca.3 (2020).

[11] Wang, M., Lu, Z., Zhao, Y. & Wan, W. Experimental and numerical study on peak strength, coalescence and failure of rock-like materials with two folded preexisting fissures. *Theoret. Appl. Fract. Mech.* **125**, 103830. 10.1016/j.tafmec.2023.103830 (2023).

[12] Wang, M., Lu, Z., Zhao, Y. & Wan, W. Peak strength, coalescence and failure processes of rock-like materials containing preexisting joints and circular holes under uniaxial compression: Experimental and numerical study. *Theoret. Appl. Fract. Mech.* **125**, 103898. 10.1016/j.tafmec.2023.103898 (2023).

[13] He, G. Q., Yang, L. & Ling, G. D. *Mining Subsidence Science* (University of Mining and Technology Press, 1991).

[14] State Bureau of Coal Industry. *Regulations on Coal Pillars Under Buildings, Water Bodies and Railways and Compressed Coal Mining* (China Coal Industry Publishing House, 2000).

[15] Knothe, S. Effect of time on formation of basin subsidence. *Arch. Mining Steel Ind.* **1**, 1–7 (1953).

[16] Xi, G. J. et al. Application of improved logistic function model to prediction of surface subsidence. *Coal Sci. Technol.* **41**, 114–117 (2013).

[17] Zhang, Y. J. et al. Dynamic prediction model of mining subsidence combined with improved Weibull time function. *Rock Soil Mech.* **45**, 1824–1834 (2024).

[18] Zhang, X. S., Yan, S. B., Tan, H. C. & Dong, J. Y. A time function-based prediction model of mining subsidence: Application to the Barapukuria coal mine, Bangla. *Sci. Rep.* **12**, 18433. 10.1038/s41598-022-23303-9 (2022). 36319670 10.1038/s41598-022-23303-9 PMC9626622

[19] Li, J. Y. & Wang, L. Mining subsidence monitoring model based on bpm-ektf and tls and its application in building mining damage assessment. *Environ. Earth Sci.* **80**, 396. 10.1007/s12665-021-09704-5 (2021).

[20] Cheng, H., Zhang, L. L., Guo, L. H., Wang, X. J. & Peng, S. L. A new dynamic prediction model for underground mining subsidence based on inverse function of unstable creep. *Adv. Civil Eng.* **1**, 136. 10.1155/2021/9922136 (2021).

[21] Piao, C. D., Zhu, B., Jiang, J. X. & Dong, Q. H. Research on prediction method of coal mining surface subsidence based on mmf optimization model. *Sci. Rep.* **14**, 20316. 10.1038/s41598-024-71434-y (2024). 39223282 10.1038/s41598-024-71434-y PMC11368931

[22] Liu, Y. C. *Study on the Dynamic Course of the Surface Subsidence and the Model Based on Theory of Key Rock Stratum*. Doctoral dissertation, Chongqing University (2010).

[23] Litwiniszyn, J. *Stochastic Methods in Mechanics of Granular Bodies* (Springer, 1974).

[24] Wu, K. et al. The technique of series prediction on mining subsidence. *Eng. Surv. Mapp.* **4**, 20–23 (2001).

[25] Gonzalez-Nicieza, C., Alvarez-Fernandez, M. I., Menendez-Diaz, A. & Alvarez-Vigil, A. E. The influence of time on subsidence in the central Asturian coalfield. *Bull. Eng. Geol. Environ.* **66**, 319–329. 10.1007/s10064-007-0085-2 (2007).

[26] Zhang, B., Cui, X. M., Zhao, Y. L. & Li, C. Y. Parameter calculation method for optimized segmented knothe time function. *J. China Coal* **43**, 3379–3386. 10.13225/j.cnki.jccs.2018.0369 (2018).

[27] He, F. S., Hu, H. F., Lian, X. G. & Zhang, K. Construction and parameter of normal time function model related to position. *J. China Coal Soc.* **45**, 766–772. 10.13225/j.cnki.jccs.2020.0555 (2020).

[28] Cui, X. M., Liao, X. X., Zhao, Y. L. & Jin, R. Discussion on the time function of time dependent surface movement. *J. China Coal Soc.* **1**, 453–456 (1999).

[29] Wu, K. A program of mining subsidence dynamic prediction and its application. *Surv. Eng.* **8**, 44–48 (1995).

[30] Hu, Q. F. et al. Model for calculating the parameter of the knothe time function based on angle of full subsidence. *Int. J. Rock Mech. Mining Sci.* **78**, 19–26. 10.1016/j.ijrmms.2015.04.022 (2015).

[31] Chen, X., Qiu, T. R., Wei, L. L. & Cai, H. Application and comparison of gm (1, 1) and metabolizing model. *Microcomput. Inf.* **24**, 163–165. 10.3969/j.issn.1008-0570.2008.12.063 (2008).

[32] Deng, J. L. *Grey System Theory Tutorial* (Huazhong University of Science and Technology Press, 1990).

